# A prospective longitudinal study evaluating the influence of immunosuppressives and other factors on COVID-19 in autoimmune rheumatic diseases

**DOI:** 10.1186/s41927-022-00264-0

**Published:** 2022-06-14

**Authors:** Abhishek Patil, K. Chanakya, Padmanabha Shenoy, S. Chandrashekara, Vikram Haridas, Sharath Kumar, Manisha Daware, Ramya Janardana, Benzeeta Pinto, Ramaswamy Subramanian, S. Nagaraj, Yogesh Preet Singh, Shweta Singhai, Ramesh Jois, Vikramraj Jain, C. Srinivasa, B. G. Dharmanand, Chethana Dharmapalaiah, K. N. Sangeetha, Vijay K. Rao, Vineeta Shobha

**Affiliations:** 1 Manipal Hospital, Bangalore, India; 2 Department of Clinical Immunology and Rheumatology, St. John’s Medical College Hospital, Sarjapur Road, Bengaluru, 560034 India; 3 CARE Hospital, Kochi, India; 4 ChanRe Rheumatology and Immunology Centre, Bangalore, India; 5 Arthritis Superspeciality Centre, Hubli, India; 6 OARC, Bangalore, India; 7 Narayana Health City, Bangalore, India; 8 JSS Medical College, Mysore, India; 9 Columbia Asia, Bangalore, India; 10 Sakra Hospital, Bangalore, India; 11 Vikram Hospital, Bangalore, India; 12 Bhagwan Mahaveer Jain Hospital, Bangalore, India; 13 Fortis Hospital, Bangalore, India; 14 Aster CMI, Bangalore, India; 15 Anagha Hospital, Mysore, India

**Keywords:** Autoimmune rheumatic diseases, SARS-CoV-2 infection, Risk factors, Outcome, Hydroxychloroquine, Glucocorticoid, Immunosuppressants

## Abstract

**Background:**

We conducted this study to identify the influence of prolonged use of hydroxychloroquine (HCQ), glucocorticoids and other immunosuppressants (IS) on occurrence and outcome of COVID-19 in patients with autoimmune rheumatic diseases (AIRDs).

**Methods:**

This was a prospective, multicenter, non-interventional longitudinal study across 15 specialist rheumatology centers. Consecutive AIRD patients on treatment with immunosuppressants were recruited and followed up longitudinally to assess parameters contributing to development of COVID-19 and its outcome.

**Results:**

COVID-19 occurred in 314 (3.45%) of 9212 AIRD patients during a median follow up of 177 (IQR 129, 219) days. Long term HCQ use had no major impact on the occurrence or the outcome of COVID-19. Glucocorticoids in moderate dose (7.5–20 mg/day) conferred higher risk (RR = 1.72) of infection. Among the IS, Mycophenolate mofetil (MMF), Cyclophosphamide (CYC) and Rituximab (RTX) use was higher in patients with COVID 19. However, the conventional risk factors such as male sex (RR = 1.51), coexistent diabetes mellitus (RR = 1.64), pre-existing lung disease (RR = 2.01) and smoking (RR = 3.32) were the major contributing risk factors for COVID-19. Thirteen patients (4.14%) died, the strongest risk factor being pre-existing lung disease (RR = 6.36, *p* = 0.01). Incidence (17.5 vs 5.3 per 1 lakh (Karnataka) and 25.3 vs 7.9 per 1 lakh (Kerala)) and case fatality (4.1% vs 1.3% (Karnataka) and 4.3% vs 0.4% (Kerala)) rate of COVID-19 was significantly higher (*p* < 0.001) compared to the general population of the corresponding geographic region.

**Conclusions:**

Immunosuppressants have a differential impact on the risk of COVID-19 occurrence in AIRD patients. Older age, males, smokers, hypertensive, diabetic and underlying lung disease contributed to higher risk. The incidence rate and the case fatality rate in AIRD patients is much higher than that in the general population.

**Supplementary Information:**

The online version contains supplementary material available at 10.1186/s41927-022-00264-0.

## Key points

1. Long term HCQ use had no major impact on the occurrence or the outcome of COVID-19 in AIRD.
2. Glucocorticoids in moderate doses increased the risk of COVID-19 in AIRD patients.
3. The incidence rate of COVID-19 is at least 3 fold higher and the case fatality rate is 4.6 times higher than that of the general population in the same geographic area.
4. The risk of contracting COVID-19 is higher in AIRD patients, who are elderly, male, smokers, hypertensive, diabetic or with an underlying lung disease.

## Introduction

The coronavirus disease (COVID-19) pandemic has affected more than 424 million persons and led to over 5.9 million deaths across the world as of 23rd February 2022 [1]. In the early part of the COVID 19 pandemic in China, it became clear that comorbidities such as diabetes (DM), hypertension (HTN), and advanced age are associated with poor outcome [2]. Patients with Autoimmune Rheumatic Disease (AIRD) have underlying immune dysfunction in addition to frequent use of glucocorticoids (GC) and other immunosuppressant (IS) medications. Hence it becomes imperative to identify the risk factors associated with Severe Acute Respiratory Syndrome Coronavirus 2 (SARS- COV2) infection and outcome in AIRD.

In addition, with the better understanding of the molecular pathways and cytokine networks in SARS-CoV2 infection, the repurposing of the existing immunomodulatory drugs to curtail the cytokine storm came to the forefront [3, 4]. Early on in the pandemic, hydroxychloroquine (HCQ) was acclaimed as both a preventive and therapeutic treatment for COVID-19, but subsequent clinical trials have not found any benefit. However, this initial euphoria led to a significant shortage of HCQ for lupus and RA patients during April-June 2020. This was mainly due to people trying to stock the drug through fake prescriptions and black markets for its perceived benefit in COVID 19 [5]. Several immunomodulatory medications including glucocorticoids and biologicals, which may potentially inhibit one or more steps of the coronavirus life cycle or can counteract the amplified immune response were focused on amongst many others [6]. In addition, there were several reports of improved outcomes of rheumatic diseases treated with TNF inhibitors compared to other immunosuppressives during COVID 19 [7].

AutoImmune Rheumatic Disease (AIRD) such as Rheumatoid arthritis (RA), Systemic lupus erythematosus (SLE), Systemic Vasculitis and several others are conventionally treated with HCQ in addition to other IS and GC. Dexamethasone has been evaluated in several clinical trials and was associated with a lower risk of mortality at 28 days in critically ill COVID-19 patients, compared to placebo or standard of care [8]. GCs form an important component of treatment for many of the AIRDs. Clinical trial protocols testing these molecules would normally exclude immunosuppressed patients [9]. Therefore, it is of significance to understand both the incidence and outcome of COVID-19 in AIRD who are already on treatment with these groups of medications.

The purpose of our study was three-fold. Firstly, through this prospective longitudinal study, we examined if in the AIRD population already on long term HCQ treatment had any impact on occurrence or severity of COVID-19. Secondly, we examined if the current use of GC and or IS would influence occurrence of COVID-19, or alter the intensity of associated hyperinflammation thereby reducing its severity in AIRD patients. Thirdly, to delineate the risk factors associated with occurrence and adverse outcome of COVID-19 in the AIRD cohort in our geographic region who were being followed up longitudinally during the 1st wave in India.

As we were writing this manuscript, India experienced the devastating effects of the second wave of COVID 19. Hence, it also becomes imperative that the real-world data should define and help derive recommendations for COVID-19 in AIRD patients.

## Methods

### Study design

This is a prospective, multicenter, non-interventional, longitudinal study involving 14 specialist rheumatology centers across Karnataka and one center in Kerala, India. Consecutive patients diagnosed with any of the AIRDs on treatment and follow-up with collaborating centers were recruited into this study from April-December 2020. The proposal was approved by respective ethics committees and written consent was obtained from all the participants.

### Data collection

Clinical information was recorded using a structured case record form (CRF) developed specifically for this study. Subsequently a virtual harmonization meeting was conducted across all the recruiting centers.

### Study population

Inclusion criteria for this study participation was a diagnosis of AIRD across all age groups. Their baseline demographics, disease subsets, current and past immunosuppressants (IS) information was recorded, with specific focus on HCQ and GC. We recorded the current dose of glucocorticoid that the patients were using at the time of enrollment. Non-immune mediated rheumatologic disorders and those not on treatment with IS and or HCQ were excluded. All comorbidities, including pre-existing lung diseases were recorded through review of their medical records. Additional medication information including use of antihypertensives such as angiotensin converting enzyme inhibitor (ACEi) or angiotensin receptor blockers (ARBs) were recorded.

### Follow-up

The patients were followed up at baseline and then at one, three and six months of initial recruitment. The data was recorded during visits to the hospital or by investigators initiated telephone calls by trained tele-callers (rheumatology nurses/ physician assistants). The information was sought regarding COVID-19 like symptoms, exposure to known COVID-19 patients and results of any COVID-19 test [Reverse Transcription-Polymerase Chain Reaction (RT-PCR) or Rapid Antigen Test (RAT)] using a checklist. Further, those who tested positive on either or both of the tests were considered as suffering from COVID-19 and their outcome information was recorded. The data collection was halted at the time of decline of the first wave of COVID-19 in India. COVID-19 testing protocols for symptomatic infection or exposed contacts were as per Government of India recommendations.

### Statistical methods

Descriptive statistics were reported as mean and SD for continuous variables, number and percentages for categorical variables. Association of COVID-19 and mortality with various characteristics of the study population was assessed using Chi square/Fisher’s Exact test and Student t test. Bivariate and multivariate log binomial regression analysis was performed to assess the predictors for COVID-19 positivity and mortality, considering the study design of prospective study (cohort study). Unadjusted and adjusted relative risk along with 95% CI were reported. Variables that showed probability value less than 0.10 in the bivariate analysis were considered for multivariate analysis by using the stepwise method. The unit for person-time for incidence computation (No. of COVID-19 cases/total person time at risk) in this study is reported as person-days which was computed as 177 days (median follow-up) for the study duration. *p* value less than 0.05 was considered statistically significant. Statistical analyses were carried out using SPSS version 25.0.

## Results

### Study population

A total of 9212 AIRD patients were recruited, the major disease subsets were RA (50.9%), SLE (15.4%), axial spondyloarthropathy (SpA) (9.1%) and psoriatic arthritis (PsA) (8%). The mean age of the cohort was 45.1 years (SD 14.3), 2% were in the pediatric age group (< 18yrs) and 77% were females. The median duration of underlying illness was 48 (22, 96) months. Their baseline characteristics, IS administered and comorbidities are detailed in Table 1. During the course of this study (median follow up 177 days; (IQR) 129, 219), 314 patients (3.4%) were diagnosed with COVID-19 based on lab confirmation (RT-PCR and/ or RAT).

**Table 1:** Comparison of clinical characteristics amongst COVID and non-COVID AIRD patients

	Overall 9212 (%)	COVID positive 314 (%)	COVID negative 8898 (%)	*p* value
Age in years	45.1 ± 14.3	46.8 ± 14.3	45.1 ± 14.2	0.028
Gender				
Male	2134 (23.2)	99 (31.5)	2035 (22.9)	< 0.001
Female	7075 (76.8)	215 (68.5)	6860 (77.1)	
Duration of AIRD in months				
1–24	2916 (32.5)	94 (30.4)	2822 (32.5)	0.658
25–48	1720 (19.2)	64 (20.7)	1656 (19.1)	
> 48	4343 (48.4)	151 (48.9)	4192 (48.4)	
Diagnosis				
RA	4558 (50.9)	120 (40.0)	4438 (51.3)	< 0.001
SLE	1379 (15.4)	36 (12.0)	1343 (15.5)	0.07
Inflammatory myositis	99 (1.1)	3 (1.0)	96 (1.1)	0.25
Systemic sclerosis	173 (1.9)	7 (2.3)	166 (1.9)	0.36
Systemic vasculitis	193 (2.2)	18 (6.1)	175 (2.0)	< 0.001
PsA	716 (8.0)	21 (7.1)	695 (8.1)	0.52
Sjogren’s	148 (1.7)	8 (2.7)	140 (1.6)	0.15
AxSpA	819 (9.1)	33 (11.1)	786 (9.1)	0.31
Sarcoidosis	58 (0.6)	4 (1.3)	54 (0.6)	0.84
Bechets	11 (0.1)	2 (0.7)	9 (0.1)	0.15
Others	720 (8.0)	45 (15.0)	675 (7.8)	
HCQ use N (%)	5266 (57.4)	167 (53.2)	5099 (57.5)	0.125
Mean dose (mg)	212 (200, 300)	200 (200, 300)	200 (200, 300)	0.136
Duration (months)	12 (3, 39)	10 (0, 38)	12 (3, 39)	0.218
Glucocorticoids N (%)	3459 (37.7)	122 (39.0)	3337 (37.7)	0.647
Mean dose mg/day (%)				
< 7.5	2652 (79.5)	91 (73.4)	2561 (79.7)	0.111
7.5–20	395 (11.8)	22 (17.7)	373 (11.6)	
> 20	289 (8.7)	11 (8.9)	278 (8.7)	
Immunosuppression				
Methotrexate	5494 (60.0)	152 (49.0)	5336 (60.3)	0.001
Azathioprine	389 (4.3)	12 (4.0)	377 (4.3)	0.800
Mycophenolate	720 (7.9)	34 (11.3)	686 (7.8)	0.029
Cyclophosphamide	58 (0.6)	8 (2.7)	50 (0.6)	< 0.001
Leflunomide	1811 (19.7)	46 (15.1)	1765 (20.1)	0.034
Tacrolimus	496 (5.5)	15 (5.0)	481 (5.5)	0.655
Rituximab	149 (1.6)	11 (3.5)	138 (1.6)	0.007
TNFi	193 (2.1)	10 (3.2)	183 (2.1)	0.170
Secukinumab	35 (0.4)	1 (0.3)	34 (0.4)	0.857
JAKinibs	21 (0.2)	0	21 (0.2)	1.000
Iguratimod	42 (0.5)	1 (0.3)	41 (0.5)	1.000
Comorbidities				
DM	993 (10.9)	62 (19.8)	931 (10.5)	< 0.0001
HTN	1385 (15.0)	68 (21.9)	1317 (14.9)	< 0.0001
Pre existing lung disease	366 (4.0)	28 (8.9)	338 (3.8)	< 0.0001
ACEi/ARBs				
Yes	898 (9.9)	39 (12.6)	859 (9.8)	0.096
No	8218 (90.1)	269 (97.1)	7841 (97.7)	
Smoking	85 (0.9)	9 (2.9)	76 (0.9)	0.002

*AIRD* autoimmune rheumatic diseases, *RA* rheumatoid arthritis, *SLE* systemic lupus erythematosus, *PsA* psoriatic arthritis, *AxSpA* axial spondyloarthritis, *HCQ* hydroxychloroquine, *TNFi* tumor necrosis factor alpha inhibitor, *JAKinibs* janus kinase inhibitors, *DM* diabetes mellitus, *HTN* hypertension, *ACEi* angiotensin converting enzyme inhibitor, *ARB* angiotensin receptor blocker

### HCQ and COVID-19

**Fig. 1:**
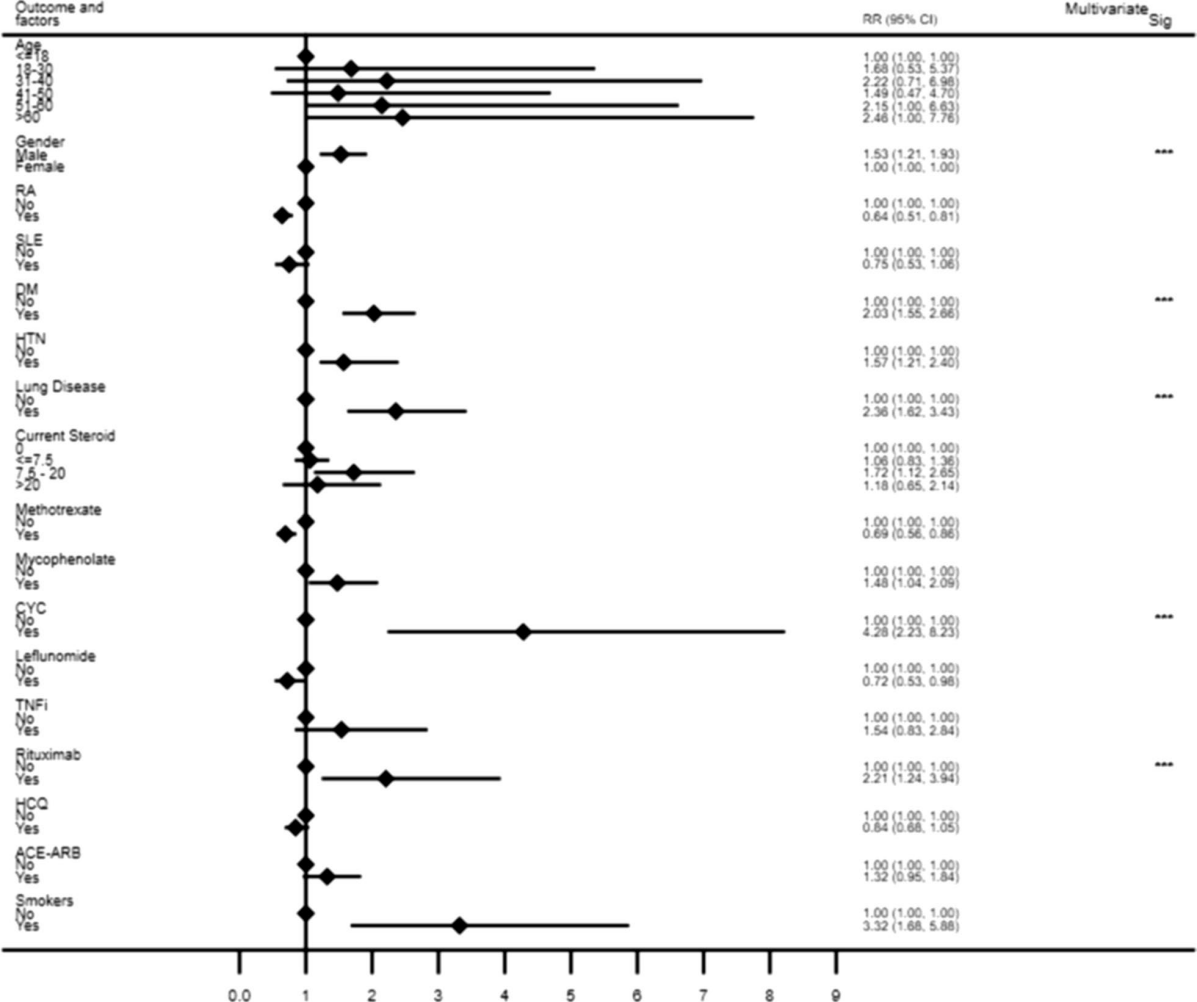
Results of bivariate analysis to assess the factors associated with the risk of COVID-19 infection. Significant values in Multivariate analysis; Gender (*p* = 0.001), DM (*p* = 0.001), Lung disease (*p* < 0.001), Glucocorticoid (7.5-20 mg) (*p* = 0.04), CYC (*p* < 0.001), Rituximab (*p* = 0.003). *Abbreviations RA* rheumatoid arthritis, *SLE* systemic lupus erythematosus, *DM* diabetes mellitus, *HTN* hypertension, *CYC* cyclophosphamide, *TNFi* tumor necrosis factor alpha inhibitor, *HCQ* hydroxychloroquine, *ACEi* angiotensin converting enzyme inhibitor, *ARB* angiotensin receptor blocker

Overall, during the study period, 57.4%, 68% and 88% of the total cohort, RA and SLE, were being treated with HCQ respectively. The mean dose of HCQ was 212 mg/day (SD 119). The median duration of HCQ use in the entire cohort was 12 months (IQR 3, 39). In the overall cohort, HCQ use did not influence occurrence of COVID-19 (RR = 0.909, CI (0.715, 1.154), *p* = 0.432) or mortality (*p* = 0.097) (Table 2, Fig. 1). In the subgroup analysis of RA and SLE, there was no independent impact of HCQ on occurrence and outcome of COVID-19.

**Table 2:** Factors associated with Mortality (N = 13) in COVID-19 population

	Unadjusted			Adjusted		
	RR	95% CI	*p* value	RR	95% CI	*p* value
Age	1.053	1.012, 1.096	0.071	1.037	0.996, 1.079	0.079
Gender M:F	1.357	0.456, 4.044	0. 583			
RA	1.475	0.487, 4.465	0.492			
SLE	0.659	0.088, 4.955	0.685			
Duration of AIRD (months)						
1–24	1					
25–48	1.606	0.534, 4.835	0.399			
> 48	0.393	0.048, 3.201	0.383			
Diabetes Mellitus	3.471	1.209, 9.960	0.021	1.623	0.519, 5.104	0.403
Hypertension	2.233	0.755, 6.607	0.146			
Pre-existing Lung involvement	6.362	2.231, 18.130	0.001	4.315	1.416, 13.150	0.010
Current Steroid use	2.23	0.87–5.71	0.09			
HCQ	0.341	0.096, 1.215	0.097			
CYC	2.41	0.36, 16.1	0.362			
MMF	1.689	0.511, 5.57	0.390			
Rituximab	1.72	0.25, 11.8	0.581			
ACEi/ARB	1.259	0.290, 5.468	0.759			
Smokers	6.162	1.593, 23.83	0.008			

Unadjusted and adjusted relative risk and 95% confidence interval using bivariate and multivariate log binomial regression analysis; Multivariate model using stepwise method—variables entered in to the model were age, gender, presence of diabetes mellitus, pre existing lung involvement, current steroid use and current HCQ use

*AIRD* autoimmune rheumatic diseases, *RA* rheumatoid arthritis, *SLE* systemic lupus erythematosus, *HCQ* hydroxychloroquine, *CYC* cyclophosphamide, *MMF* mycophenolate mofetil, *ACEi* angiotensin converting enzyme inhibitor, *ARB* angiotensin receptor blocker

### GC and COVID-19 (Table1, Fig. 1)

In the entire cohort, 3459 (37.7%) AIRD patients were using GC at the time of participation in this study. The use of GC was analysed in 3 groups viz. < 7.5 mg, 7.5-20 mg, and > 20 mg, almost four fifths (79.1%) of them were using a dose < 7.5 mg/day. In the univariate analysis, those on GC moderate dose category (7.5–20 mg), had the highest risk (RR = 1.72, CI (1.12, 2.64), *p* = 0.01) of COVID-19, which was substantiated in multivariate analysis (RR 1.57, CI (1.003, 2.47), *p* = 0.048). Current use of steroids trended towards moderate influence on mortality as well (RR 2.23, CI (0.87–5.71) *p* = 0.09) (Table2).

### Associations with other IS, biologics

A higher association of COVID-19 positivity was noted with the use of cyclophosphamide (CYC) (*p* < 0.001) and mycophenolate (MMF) (*p* = 0.029) (Table1). Amongst those on treatment with biologics, risk was higher with rituximab (RTX) (*p* = 0.007). In the multivariate analysis, CYC [RR-4.2, CI (2.23, 7.91) *p* < 0.001] and RTX [RR 2.4, CI 1.35–4.32 *p* = 0.003] use were independent risk factors associated with increased risk of COVID-19 (Fig. 1, Additional file 1: Table S1). However, MMF (*p* = 0.39), CYC (*p* = 0.36) and RTX (*p* = 0.58) use did not affect mortality (Table 2).

### Disease subsets

We found that a lesser proportion of RA patients developed COVID-19 compared to other AIRDs (RR-0.642, CI 0.51, 0.81, *p* = 0.041). Patients with systemic vasculitis had the highest risk of contracting COVID-19 (*p* < 0.001) (Table 1).

### Comorbidities and other parameters

The parameters associated with COVID-19 positivity were older age (*p* = 0.025), male sex (*p* = 0.001), smoking (*p* = 0.002), comorbidities such as DM (*p* < 0.0001), HTN (*p* < 0.0001), presence of underlying lung disease such as interstitial lung diseases, asthma, chronic obstructive pulmonary disease (*p* < 0.0001) and smoking (*p* = 0.002). The relative risk of occurrence of COVID-19 with 95% CI is detailed in Fig. 1 (Additional file 1: Table S1). Multivariate analysis adjusted for age, gender, DM, HTN, lung involvement and immunosupression use, it was noted that male sex [RR-1.51, *p* = 0.001], the coexistent DM [RR-1.64, *p* = 0.001], underlying lung disease [RR-2.01, *p* < 0.0001], and smoking [RR-3.32, *p* < 0.0001] were the independent risk factors associated with the increased risk of COVID-19. Use of ACEi/ARBs had no significant impact on occurrence or outcome.

### Outcome of COVID-19

Among the 314 COVID-19 patients, 13 (4.14%) died. The factors associated with death are presented in Table 2. The pre-existing lung disease was the strongest risk factor associated with increased risk for mortality (RR 4.315, CI (1.416, 13.15) *p* < 0.01) apart from conventional risk factors such as smoking and coexisting DM.

### Comparison of incidence and mortality of COVID-19 with general population

The incidence of COVID-19 in our cohort was significantly higher compared to the incidence in the general population (*p* < 0.0001) both in Karnataka and Kerala as depicted in Table 3. Case fatality rate was 4.5 times higher (4.1% in AIRD cohort vs 0.9% in the general population) amongst the AIRD population.

**Table 3:** Comparison of COVID-19 incidence and mortality in AIRD vs general population

	Karnataka		Kerala	
	AIRD cohort	General population	AIRD cohort	General population
Incidence rate	17.5/100,000 population	5.3/100,000 population	25.3/100,000 population	7.9/100,000 population
Case fatality rate (%)	9/221	12,080/918,473	4/93	3070/760,692
	4.1%	1.31%	4.3%	0.4%

Incidence rates and mortality (*p* < 0.001), significantly different between Karnataka and Kerala states (both AIRD and general population)

## Discussion

This longitudinal prospective study in AIRD patients was envisaged and initiated before we knew about differential risk factors for SARS-CoV2 infection and the impact of immunomodulatory therapies on its occurrence and outcome. Our AIRD cohort consists of a population from Karnataka and Kerala, who were followed up for approximately 6 months paralleling the time period of 1st peak of COVID-19 in our country. The incidence rate of COVID-19 was threefold higher in our cohort; as compared to the general population in the same region (Table 3). Wang et al. in systematic meta-analysis which included data from 26 studies and about 2000 patients, reported 1.5 times higher risk for COVID-19 in rheumatic patients (OR = 1.53, 95% CI 1.24–1.88) [10] In our cohort of more than 9000 AIRD patients, RR is more than 3 times that of the general population.

Many of AIRD such as RA and SLE are treated routinely with HCQ, whereas others such as SpA, PsA and primary vasculitis are not. This brings out a natural selection of a cohort with and without the HCQ in AIRD, and allows us to assess the differential effect of this drug on occurrence as well as outcome of COVID-19. After adjusting for confounding variables, in our AIRD cohort, long term HCQ use did not influence either occurrence or mortality of COVID-19. The role of HCQ in COVID-19 prevention and management has been greatly debated with several authors reporting conflicting outcomes [11–13]. The potential beneficial role of HCQ in terms of preventing viral entry and replication may be fractional and its immunomodulatory effect could possibly be offset by the interaction with antiviral drugs in the acutely sick patients [14]. Through our study, we could not substantiate its role as a prophylactic immunomodulator despite its use for many months or years. Hence, we emphasize the use of HCQ in AIRD based on its need for the underlying rheumatic disease and not with respect to COVID-19 pandemic.

In our cohort, we found an increased rate of infections with GC in moderate doses of 7.5–20 mg of prednisolone equivalent per day (RR 1.57) and not in lower dose (< 7.5 mg/day). Glucocorticoids have been consistently reported to increase the risk of both opportunistic and serious infections in AIRD [15]. However, in COVID-19 related hyperinflammation they have therapeutic value. Favalli et al. demonstrated a higher risk of COVID-19 in patients with AIRD, even at doses less than 2.5 mg/day of prednisolone (OR of 2.89) [16]. In its initial report of over 600 cases of COVID-19 in AIRD, Global rheumatology analysis network found a two times of odds of hospitalization with the use of steroids of > 10 mg prednisolone /day [17]. The same group identified the current GC usage of (> 10 mg/day prednisolone) to be associated with higher mortality [18]. Marques et al. demonstrated GC use to be associated with unfavorable outcomes of COVID-19 in rheumatic diseases [19]. However, this increased morbidity and mortality associated with GC use could be the result of higher disease activity in patients with higher dose of steroids rather the direct association [20]. With the demonstration of significant mortality benefit by the use of Dexamethasone in RECOVERY trial there has been renewed interest in the use of steroids in COVID-19 [8]. In our study, we could not determine decisively the impact of steroids on mortality though there was a trend towards it. While the acute short term use of steroids might help to improve the outcomes in moderate- severe COVID 19 by curtailing the cytokine storm, long term use may lead to increased susceptibility to infections and prothrombotic state which could also explain the poorer outcome of COVID 19 in patients on long term steroids. In line with the American College of Rheumatology recommendations, we advocate minimization of steroid use in AIRD to the lowest possible dose and shortest duration wherever feasible till further conclusive date becomes available [21].

Amongst other ISs, MMF and CYC confer substantially higher risk of COVID-19, while other IS/csDMARDS did not have any major impact. Scire and colleagues from Italy; also found no significant influence of csDMARDs on the risk of hospitalization or mortality {OR 0.54 [CI 0.22–1.37] *p* = 0.188} [22]. Overall, the effect of csDMARDs on COVID-19 risk appears to be minimal. At the very least, appropriate use of csDMARDs for the underlying AIRD must not be withheld in the wake of COVID-19 pandemic.

Among the biologicals and targeted synthetic DMARDs (tsDMARDS), RTX exposure was greater among the COVID-19 patients in our cohort (3.5% vs 1.6%) (Table1). In a French cohort, RTX use was associated with the higher risk of severe COVID-19 disease (RR 3·26) [23]. Pablos et al. from Spain reported a higher incidence of COVID-19 in AIRD treated with bDMRADs/ tsDMARDs {OR 1.60 95% CI (1.23–2.10)} but not in those with cDMARDs [24]. Sparks and colleagues analyzed 2869 RA patients and found RTX and JAK inhibitors to be associated with worse outcomes compared to those on TNFi [25]. Interestingly, at least 3 reports from different parts of the world describe a reduction in risk of severe COVID-19 with the use of bDMARDs particularly TNFi [22, 26, 27]. There was no significant influence of TNFi or secukinumab use in our cohort. Because of small numbers and the differential dosing regimens of bDAMRDs, we can not derive any conclusion on their exact role in the outcome or occurrence of COVID-19. Similarly, the tsDMARDs did not influence the occurrence or outcome of COVID-19 in our cohort.

RA was the most common disease subset in our cohort, being the most common AIRD in the community. Patients with systemic vasculitis had the highest risk of contracting COVID-19 (18/189; 9.5%), while RA had a lower risk (2.6%). This could be related to the use of higher doses of steroids and more intense IS in systemic vasculitis compared to other disease subsets.

Most important risk factors for developing COVID-19 in our cohort were older age, male sex smoking, underlying comorbidities such as DM, HTN and pre-existent lung disease. Early on in this pandemic, it was understood that DM and HTN form the major risk for COVID-19 [28, 29]. Bhandari et al. reported HTN and DM to be the major underlying conditions from Jaipur, India in 522 COVID-19 patients [30]. Pre-existing lung disease and smoking carried the highest risk of COVID-19 related mortality in our cohort. This is similar to other cohorts of COVID-19 in rheumatic diseases where DM, HTN, age > 65 years and pre-existent lung disease were responsible for poor outcome [31]. A recent Brazilian study of a cohort of AIRD also found a higher requirement for emergency care in diabetic patients compared to non-diabetics (OR 1.38; *p* = 0.004) [19]. A recent meta-analysis also confirmed HTN (OR = 3.69, 95% CI 1.41–9.69, *p* = 0.008) and lung disease (OR = 2.93, 95% CI 1.64–5.23, *p* = 0.000) to predict hospitalization risk [10]. Therefore, the risk factors which increase the susceptibility to COVID-19 and adverse outcome in the general population hold true even in patients with AIRD.

Gender bias among patients with COVID-19 is a globally documented phenomenon. It is postulated to be the effect of sex hormones, stronger interferon response, higher helper & cytotoxic activity of T cells, differential ACE2 expression and several others [32, 33]. In our COVID-19 infected cohort too, there was a preponderance of males in the multivariate analysis (RR = 1.51), however it did not influence mortality. Even though the number of smokers was relatively small in our cohort (< 1%), association with the COVID-19 disease was quite noteworthy (*p* = 0.002). Leung et al. demonstrated increased ACE2 gene expression in the bronchoalveolar lavage samples of smokers versus never-smokers to be the reason for increased susceptibility [34]. However, the data from China and Italy indicated a lower risk of COVID-19 and severity in current smokers [35, 36]. On the contrary, a large UK study involving more than 2.4 M participants has demonstrated a higher risk of COVID-19 in current smokers (OR 1.14) [37].

Initial concerns regarding the use of ACEi/ ARBs surfaced due to the upregulation of ACE 2 receptors on epithelial cells which are a portal of entry for SARS-COV-2 [38, 39]. However, a meta-analysis of 10 studies, found neither the risk of COVID-19 nor severity of infection to be increased with the use of ACEi/ ARBs [40]. In our cohort of patients with AIRD, we found no significant impact of ACEi/ ARB use on the risk of COVID-19 occurrence or mortality.

Case fatality rate in our cohort was 4.14% which is 4.6 times higher than that in the general population from the same geographic area (0.9%). OpenSAFELY- an initiative by the NHS, analysed the risk factors associated with the occurrence of COVID-19 in more than 1.7 Million people in England [41]. They found a higher risk of COVID-19 related death (OR of 1.3) in patients with RA, SLE and Psoriasis. In another systematic review and meta-analysis, the fatality rate was 7% in the entire analysis and 6.7% in the GRA cohort, both significantly higher than the WHO database (3.4%) [42]. In our cohort, the strongest risk factor associated with mortality was underlying lung disease. Furthermore, the disease subsets, immunosuppressants and other comorbidities did not have much influence on mortality in our cohort.

To the best of our knowledge this is the first longitudinal cohort of impact of COVID-19 on any immunocompromised or immunodeficient cohorts from India. Initial results of this cohort were published during the early part of the pandemic [43]. Strengths of our study are its prospective longitudinal non-interventional design, large sample size from specialist rheumatology centres, inclusion of RT-PCR or RAT confirmed COVID-19 patients and investigator initiated follow-up telecalls. Limitations of our study include inability to accurately assess the impact of disease activity on occurrence and outcome of COVID-19. As rheumatologists are not the primary physicians for COVID-19 care, we were not able to access precise information with regards to O2 therapy, hospitalization and ICU care. Also, the testing for COVID-19 in individual patients was as per Govt. of India, Govt. of Karnataka and Govt. of Kerala protocols which have undergone modifications as the pandemic progressed. Our data may not have captured all asymptomatically infected patients in this analysis. This could have biased the result and might have altered the reported incidence as well as mortality. Also the comparison of incidence and mortality of COVID-19 in AIRD with the general population might have been influenced by the differential age and sex composition in both the populations.

## Conclusions

Long term HCQ use had no significant impact on COVID-19 occurrence and mortality in AIRD patients while moderate doses of GC increased the risk of infection. AIRD patients who are elderly, male, smokers, hypertensive, diabetic and those with underlying lung disease have a higher risk of contracting COVID-19. The incidence rate is at least threefold higher and the case fatality rate is 4.6 times higher than that of the general population in the same geographic region during the same time period. Hence, this group of AIRD regardless of age and other comorbidities, merits first access to the various protective measures implemented against COVID-19.

## Supplementary Information

**Additional file 1:** Results of bivariate and multivariate analysis to assess the factors associated with the risk of COVID-19.

## Data Availability

The data and materials are available from all authors.
