# Supplementary material for: A prospective longitudinal study evaluating the influence of immunosuppressives and other factors on COVID-19 in autoimmune rheumatic diseases
Source: BMC Rheumatol. 2022 Jun 14;6:32. doi: 10.1186/s41927-022-00264-0 (PMC9192133; doi:10.1186/s41927-022-00264-0)
Supplement: Supplementary file 1 — Additional file 1: Results of bivariate and multivariate analysis to assess the factors associated with the risk of COVID-19. [file 41927_2022_264_MOESM1_ESM.docx]

**Supplementary Table 1: Results of bivariate and multivariate analysis to assess the factors associated with the risk of COVID-19 infection**

|  | Unadjusted | | |  | Adjusted* | | |
| --- | --- | --- | --- | --- | --- | --- | --- |
|  | RR | 95% C.I. | P value |  | RR | 95% C.I. | P value |
| Age | 1.008 | 1.001, 1.016 | **0.025** |  | 1.006 | 0.99,1.01 | 0.198 |
| Gender  (Male vs female) | 1.527 | 1.209, 1.927 | **<0.0001** |  | 1.51 | 1.17,1.94 | **0.001** |
| RA | 0.642 | 0.511, 0.806 | **0.041** |  |  |  |  |
| SLE | 0.750 | 0.532, 1.057 | 0.100 |  |  |  |  |
| Duration of AIRD  1-24 mo  25-48 mo  >48 mo | 1  1.154  1.079 | 0.845, 1.577  0.837, 1.389 | 0.367  0.558 |  | 1.02  1.08 | 0.73,1.42  0.82,1.40 | 0.88  0.58 |
| Diabetes Mellitus | 2.028 | 1.548, 2.657 | **<0.0001** |  | 1.64 | 1.21,2.22 | **0.001** |
| Hypertension | 1.570 | 1.207, 2.401 | **<0.0001** |  | 1.19 | 0.89,1.59 | 0.23 |
| Pre-existing Lung involvement | 2.358 | 1.622, 3.426 | **<0.0001** |  | 2.01 | 1.36,2.96 | **<0.001** |
| Current Steroid use | 1.05 | 0.84,1.32 | 0.647 |  | 1.003 | 0787, 1.277 | 0.983 |
| No  <=7.5mg/d  7.5-20 mg/d  >20 mg/d | 1  1.06  1.72  1.17 | 0.83.1.35  1.12,2.64  0.65,2.13 | 0.64  **0.01**  0.59 |  | 1  0.96  1.57  1.09 | 0.73,1.26  1.003,2.47  0.58,2.04 | 0.79  **0.048**  0.78 |
| Methotrexate | 0.692 | 0.556, 0.861 | **0.001** |  |  |  |  |
| Mycophenolate | 1.475 | 1.041, 2.091 | 0.029 |  | 1.387 | 0.957, 2.010 | 0.084 |
| Leflunomide | 0.718 | 0.527, 0.978 | 0.035 |  |  |  |  |
| CYC | 4.28 | 2.23, 8.23 | **<0.001** |  | 4.20 | 2.23, 7.91 | **<0.001** |
| TNFi | 1.537 | 0.832, 2.839 | 0.170 |  |  |  |  |
| Rituximab | 2.208 | 1.237, 3.942 | **0.007** |  | 2.42 | 1.35, 4.32 | **0.003** |
| HCQ | 0.844 | 0.678, 1.049 | 0.126 |  | 0.909 | 0.715, 1.154 | 0.432 |
| ACEi/ARB | 1.322 | 0.952, 1.836 | 0.096 |  |  |  |  |
| Smokers | 3.318 | 1.675, 5.877 | **<0.0001** |  |  |  |  |

Abbreviations: AIRD-Autoimmune rheumatic diseases, RA- rheumatoid arthritis, SLE- systemic lupus erythematosus, CYC- cyclophosphamide, HCQ-hydroxychloroquine, TNFi- tumor necrosis factor alpha inhibitor, ACEi- angiotensin converting enzyme inhibitor, ARB- angiotensin receptor blocker

*P value using log binomial regression analysis; RR - relative risk; 95%C.I.- 95% confidence interval; HCQ use adjusted for age, gender, Diabetes Mellitus, Hypertension, Lung involvement, Cyclophosphamide, Mycophenolate, Rituximab, Current steroid dose, duration of AIRD
